# Connecting the Dots: The Role of Pediatric Concussion on Pubertal Hormones and Psychological Health

**DOI:** 10.1016/j.jadohealth.2025.07.020

**Published:** 2025-09-16

**Authors:** João Paulo Lima Santos, Elizabeth A. Shirtcliff, Anthony P. Kontos, Cecile D. Ladouceur, Amelia Versace

**Affiliations:** a Department of Psychiatry, University of Pittsburgh, Pittsburgh, Pennsylvania; b Department of Psychology, University of Oregon, Eugene, Oregon; c Department of Orthopaedic Surgery, University of Pittsburgh, Pittsburgh, Pennsylvania; d Magnetic Resonance Research Center, University of Pittsburgh, Pittsburgh, Pennsylvania

**Keywords:** Concussion, Puberty, Pediatrics, Adolescents, Hormones, Psychological health, Mental health

## Abstract

**Purpose:**

Psychological health issues resulting from emotional dysregulation such as anxiety and depression following pediatric concussion are a public health concern, but the underlying mechanisms remain unclear. Puberty opens a window of vulnerability for emotion dysregulation and a link between pediatric concussion and hormonal deficits has been reported. We investigated the association between pubertal hormones and psychological health in children with and without concussion history.

**Methods:**

Participants included children (9–10 years) from the Adolescent Brain Cognitive Development study comprising with and without a reported history of concussion within the previous 2 years. Salivary Dehydroepiandrosterone (DHEA), testosterone and estradiol were used to determine levels of pubertal hormones, and the Child Behavior Checklist scores were used to characterize psychological health issues in late childhood (N = 264; 50% concussion history; 9.99 [0.64]yy; 36% female) and early adolescence (N = 112; 48% concussion history; 12.06 [0.66]yy; 33% female). Analyses of covariance were performed to relate concussion history to hormone levels from late childhood to early adolescence. Additional analyses determined whether hormone levels at late childhood or changes between late childhood and early adolescence mediated the effect of concussion history on psychological health.

**Results:**

Children with a concussion history had lower testosterone at late childhood (F = 6.5, *p* = .012, Q = 0.035) and larger DHEA increases over time (F = 6.0, *p* = .016, Q = 0.048). Larger DHEA increases partially mediated the association between concussion history and Child Behavior Checklist scores.

**Discussion:**

Pediatric concussion may increase vulnerability to psychological health issues in adolescence via pubertal hormones. Longitudinal studies evaluating the impact of pediatric concussion on psychological health and hormones are warranted to clarify this potential vulnerability.

Concussion heightens the risk for psychological issues and raises major public health concerns [1–3], especially in pediatric samples [4]. A recent population-based retrospective study including over 400,000 children and adolescents indicated that those with a history of concussion (N = 152,321) had a 40% higher risk for hospitalizations and psychological health issues (171,563) [1], including depression and anxiety. Despite the growing recognition of the problem, the mechanisms underlying this risk remain unclear. Our recent work indicates that pubertal maturation has an impact on emotional regulation brain regions after pediatric concussion, with adolescents who are more advanced in puberty at the time of injury being more vulnerable in these regions than those who have not yet entered puberty [5]. While prior studies have demonstrated structural and functional impairments in the hypothalamus and pituitary gland—regions critical for regulating pubertal development—following mild to severe brain injuries in adults [6–8], the impact of concussion on pubertal hormones, a key driver of pubertal development, remains poorly understood. Investigating this association is crucial for a better understanding of how concussion relates to psychological health.

Puberty is a developmental period marked by physical, sexual, and social changes driven by the rise of pubertal hormones (e.g., dehydroepiandrosterone (DHEA); testosterone; E2), with adrenarche typically onsetting before gonadarche [9–12]. These hormones trigger the expression of genes that exert micro and macro structural brain changes [10–12], prominent in regions involved in higher cognitive control functions, such as emotion regulation [13,14]. Evidence from animal models indicates that pubertal hormones may affect how the brain responds to concussion, with E2, progesterone, and DHEA exerting neuroprotective effects [15,16]. However, while recent reviews have shown that concussion may be associated with growth hormone deficits in humans [17,18], it remains unclear whether concussion affects pubertal hormones. Understanding this association is of utmost importance for those children entering puberty, when the normative development of brain regions involved with emotional regulation is strongly influenced by the rise of pubertal hormones [19,20]. Therefore, it is conceivable that pubertal hormonal deficits following a concussion could disrupt the normative development of these regions in children transitioning from late childhood to early adolescence, potentially leading to a greater risk for psychological issues even later in life [21]. Thus, the relationship between concussion and psychological issues could be in part explained by the effects of concussion on pubertal hormones. Understanding these relationships could clarify how concussion influences pubertal hormones and psychological outcomes in adolescents. Large, diverse, and longitudinal studies are essential to uncover the mechanisms linking concussion, hormone changes, and psychological symptoms over time.

By leveraging an already-existing longitudinal dataset collected by the Adolescent Brain Cognitive Development (ABCD) study [22,23], we sought to investigate the association between concussion and salivary levels of pubertal hormones (DHEA, testosterone, and E2) in late childhood, and hormonal changes between late childhood and early adolescence (2 years later). Additionally, we aimed to further determine if these concussion-related effects on hormones mediate the relationship between concussion and psychological health issues reported in early adolescence. We hypothesized that (1) history of concussion (within 2 years prior to study entry) would be associated with lower levels of pubertal hormones in late childhood, and with smaller increases from late childhood to early adolescence in comparison to a matched sample of youth with no history of concussion. We also hypothesized that (2) these concussion-related effects would partially mediate the relationship between history of concussion and psychological issues. We explored whether sex moderated the effects of concussion on pubertal hormones.

## Methods

### Participants

In the ABCD study, parents and guardians provided written informed consent and children provided assent. Study-entry and 2-year follow-up data (5.1 release) available in the National Institute of Mental Health Data Archive were used to identify participants with or without concussion history in the 2 years prior to the ABCD study-entry. Concussion history was based on parental reports in the Ohio State University traumatic brain injury (TBI) Screen-Short [24] at study-entry, including history of potential head injuries, but also associated signs or symptoms (e.g., loss of consciousness, post-injury amnesia) and age at injury. The Ohio State University TBI Screen-Short identifies five categories: improbable TBI, possible concussion, Concussion, moderate TBI, and severe TBI.

To test our hypotheses, participants were clustered into two groups: previously concussed or nonconcussed. As previously done [25], we adopted an operationalization strategy, whereby participants categorized as ‘possible concussion’ or ‘concussion’ were included in the concussed group, whereas those categorized as ‘improbable TBI’ were included in the nonconcussed group (see the flowchart in Figure S1 for inclusion/exclusion criteria). A total of 132 adolescents had a concussion reported in the 2 years prior to study-entry. Given the wide range of demographic and hormone collection characteristics, a subsample of matched 132 controls was selected from those who classified as nonconcussed (N = 3,681) using the *matchit* package in R [26]. This selection maximalized the matching for biologically relevant variables (age, sex, puberty, race/ethnicity, and body mass index) to (a) minimize their confounding effects and (b) focus on the effects of history of concussion on our variables of interest. In the early adolescence follow-up (2 years later), 112 participants (54 concussed and 58 nonconcussed) had both clinical and hormonal data available. Table 1 shows clinical and demographic characteristics of all samples.

### Hormonal and pubertal data

Pubertal hormones were measured from saliva provided by participants at both visits. In the ABCD study, pubertal hormones were assessed through the collection of a single salivary biospecimen via passive drool at each time point [27,28]. Briefly, participants avoided food, drinks, gum, or mints for 30 minutes, major meals for 60 minutes, and rinsed their mouths with water 10 minutes before saliva collection. Samples were collected between 7:00 a.m. and 7:00 p.m., stored in chilled coolers, and later transferred to on-site freezers. Saliva samples were shipped on dry ice within 2–6 months for hormone analysis (testosterone, DHEA, and E2) using validated saliva immunoassays. Hormones were assayed in duplicate on a single day to avoid freeze-thaw cycles [27,28]. Cleaning procedures for the hormonal data were implemented as recommended by Herting et al. [28]. Participants who matched biological sex at birth and sexual identity at study-entry were included in the analyses. However, those missing data and/or whose salivary measures lied 2SD above/below the mean (outliers) were excluded [28]. Sample collection variables such as time of specimen collection (minutes since midnight), duration of specimen collection (minutes), time between specimen collection and freezer storage (minutes), intake of caffeine (yes/no), and vigorous physical exercise (yes/no) were also collected [27]. Stability of collection time across follow-ups is also particularly important in longitudinal analyses involving hormones [27]. For the analysis, z-scores of testosterone and DHEA were derived for boys and girls separately.

Pubertal status was assessed using the Pubertal Development Scale (PDS) [29] and a PDS score for overall pubertal status was derived using the coding system developed by Shirtcliff et al. (ranging from 1 to 5) [30]. To account for the unique contribution of puberty on developmental models, new developmental approaches recommend ‘factoring out’ the age-PDS shared variance [31]. Therefore, we regressed out age-related effects from PDS scores in males and females separately to derive pubertal timing for the analyses. Scores for adrenarcheal (PDSA) and gonadarcheal (PDSG) development were also calculated. PDSA scores included indicators of pubic/body hair and skin changes for both sexes. For PDSG scores, the indicators included growth spurt, breast development, and menarche in girls, and growth spurt, voice deepening, and facial hair growth in boys [29,30].

### Psychological health measures

The Child Behavior Checklist (CBCL) [32] is a parent-report questionnaire with 113 questions that cluster into two higher-order factor scores characterizing internalizing (psychological: anxious/depressed, withdrawn/depressed, and somatic complaints) and externalizing problems (rule-breaking and aggressive behaviors). The CBCL was collected at each visit. Each question is scored using a three-point Likert scale (0 = absent,1 = occurs sometimes, 2 = occurs often). Total scores for internalizing problems served as main outcome, those for externalizing problems served for exploratory analyses.

### Additional data

Information regarding age, sex assigned at birth, pubertal status, household income, parent education, and race was also available (see Supplementary Materials for additional information).

### Statistical approach

Analyses of covariance were used to test our first hypothesis and thus identify the association between history of concussion and *i*. levels of pubertal hormones at late childhood and *ii*. changes between late childhood and early adolescence. A regularized multivariate mediation analysis was used to test our second hypothesis, and thus identify possible mediators of the concussion-psychological issues relationship. The *matchit* package minimized between-group differences in biologically relevant variables. To maximize power, covariates included clinical and demographic characteristics that differed between groups (Table 1) and ABCD site (Figure S2). False discovery rate [33] was used to account for multiple testing as appropriate.

#### Analysis 1: Effects of concussion on late childhood pubertal hormones and 2-year changes.

In the three analyses of covariance using hormone levels in late childhood as dependent continuous variable (one for each hormone), history of concussion served as independent variable.

For participants with data available at the follow-up, we evaluated the effects of concussion on hormonal changes. Here, hormonal changes (delta: 2-year levels subtracted by late childhood levels) served as dependent variable, covarying for late childhood levels for each respective hormone.

Of note, the effects of history of concussion on E2 measures were not examined in male participants because indices are reliable only in female participants [34].

#### Analysis 2: Mediation effects.

The R package *mma* [35] was used to identify possible mediators between exposure (history of concussion) and outcome (psychological issues). Bootstrapping models (10,000 resamples) were performed using PROCESS macro [36]. Two sets of analyses were performed to include possible mediators as described below.

- Possible mediators in analysis using psychological issues in late childhood as outcome included hormonal levels that in late childhood resulted to be affected by a history of concussion (Analysis 1).
- Possible mediators of analysis using psychological issues in early adolescence (2-year follow-up) as outcome included hormonal levels that in late childhood, and/or 2-year changes, resulted to be affected by a history of concussion (Analysis 1). Here, psychological issues (CBCL) in late childhood served as covariate.

### Exploratory analyses

#### Contribution of demographic variables.

Analyses re-examined the effects of concussion on hormones proposed in Analysis 1 after including all demographic variables (Table 1) as covariates.

#### Moderation effects.

We used PROCESS macro to explore the moderation effect of age, sex at birth, and pubertal timing on the effects of history of concussion on pubertal hormones (DHEA and testosterone) and psychological issues.

#### Externalizing problems.

Additional analyses re-examined Analysis 2 using externalizing problems as alternative outcome.

#### Indirect effects on pubertal maturation.

To determine if concussion-related changes in pubertal hormones affect pubertal maturation, for any significant finding in Analysis 1, we performed mediation analyses with pubertal maturation (PDSA or PDSG) as dependent variable (one at the time), history of concussion as independent variable, and pubertal hormone affected by concussion as mediator (DHEA for PDSA and testosterone or estradiol for PDSG, including either hormonal levels at late childhood or changes at early adolescence).

#### Recency.

Pearson correlations evaluated whether the interval time between the date of concussion in the previous 2-year and study-entry (in days) had a significant effect on hormone levels affected by concussion.

## Results

### Characteristics of the sample

In the late childhood sample (N = 264), participants with history of concussion showed higher parent education (x^2^ = 5.28,*p* = .022) than those without history of concussion (Table 1). There was no other demographic difference between groups. Regardless of history of concussion, female participants showed higher levels of testosterone and DHEA than boys (Table S1).

In the early adolescence sample (N = 112), there were no between-group differences. At each time point, there was no difference in sample collection variables. In those with no history of concussion, female participants showed higher testosterone than boys (Table S1).

Additionally, collection time across time points was stable (no differences between visits; F = 0.9, *p* = .331).

### Effects of concussion on late childhood pubertal hormones and 2-year changes

**Figure 1.**
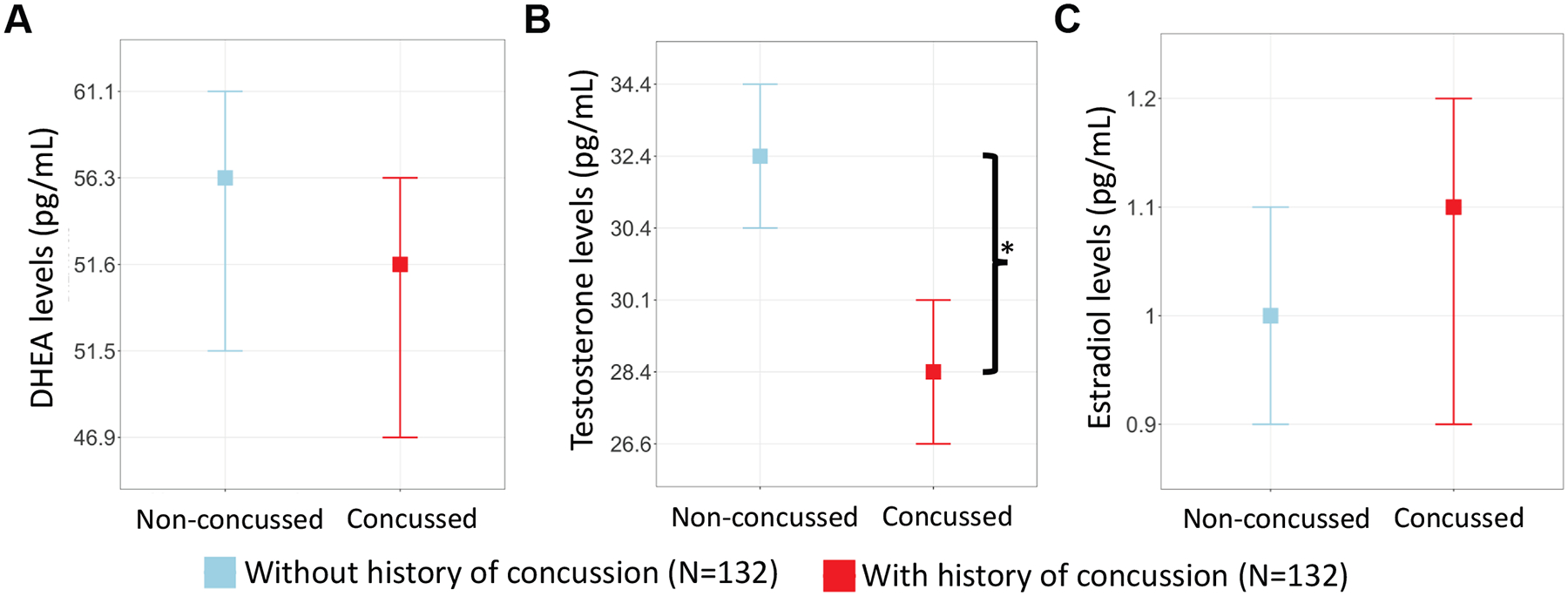
Effect of history of concussion on levels of pubertal hormones in late childhood. The figure shows participants with (N = 132, in *red color*) and without history of concussion (N = 132, in light *blue color*) levels of each pubertal hormone as follows: panel (A) DHEA; panel (B) testosterone; and panel (C) estradiol. Braces and asterisks indicate *p* values that survived FDR correction. DHEA = Dehydroepiandrosterone; FDR = false discovery rate.

In the late childhood sample (N = 264), having a history of concussion was associated with lower levels of testosterone (Figure 1) across sexes. There were no effects on DHEA or E2 (Table 2).

**Figure 2.**
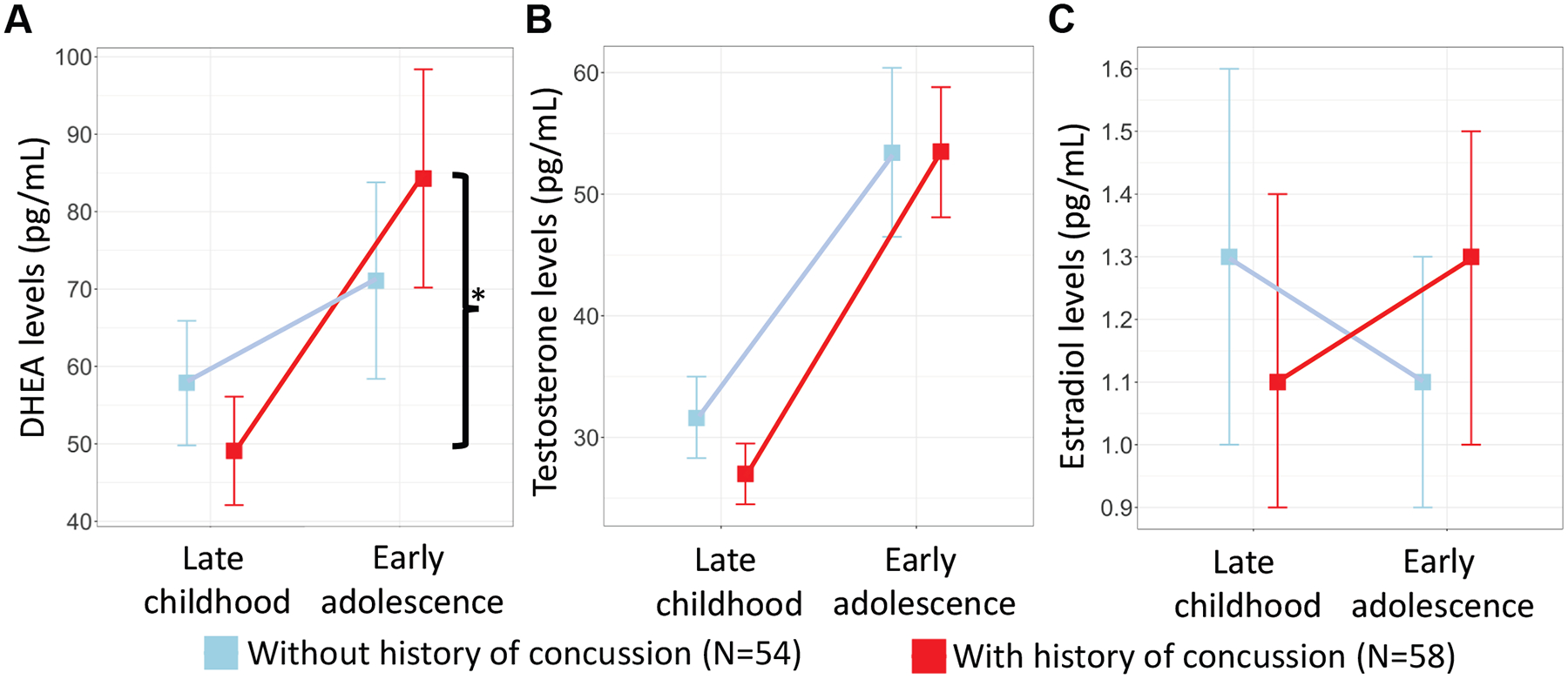
Effect of history of concussion on 2-year changes of pubertal hormones between late childhood and early adolescence. The figure shows levels of each pubertal hormone in participants with and without history of concussion in late childhood and early adolescence as follows: panel (A) DHEA; panel (B)testosterone; and panel (C) estradiol. Braces and asterisks indicate *p* values that survived FDR correction. Age, sex at birth, and pubertal timing had no moderation effect on these relationships. DHEA = dehydroepiandrosterone; FDR = false discovery rate.

In those with early adolescence data (N = 112), after accounting for late childhood levels of DHEA, history of concussion was associated with larger 2-year increases in DHEA relative to those without history of concussion (Figure 2). There were no effects on 2-year changes of testosterone or E2.

### Mediation effects

#### Psychological issues in late childhood (N = 264).

History of concussion was associated with more severe psychological issues (F_[1,257]_ = 34.08,*p* < .001,Q < 0.001) in late childhood. No hormone was selected as mediators of this association.

#### Psychological issues in early adolescence (N = 112).

**Figure 3.**
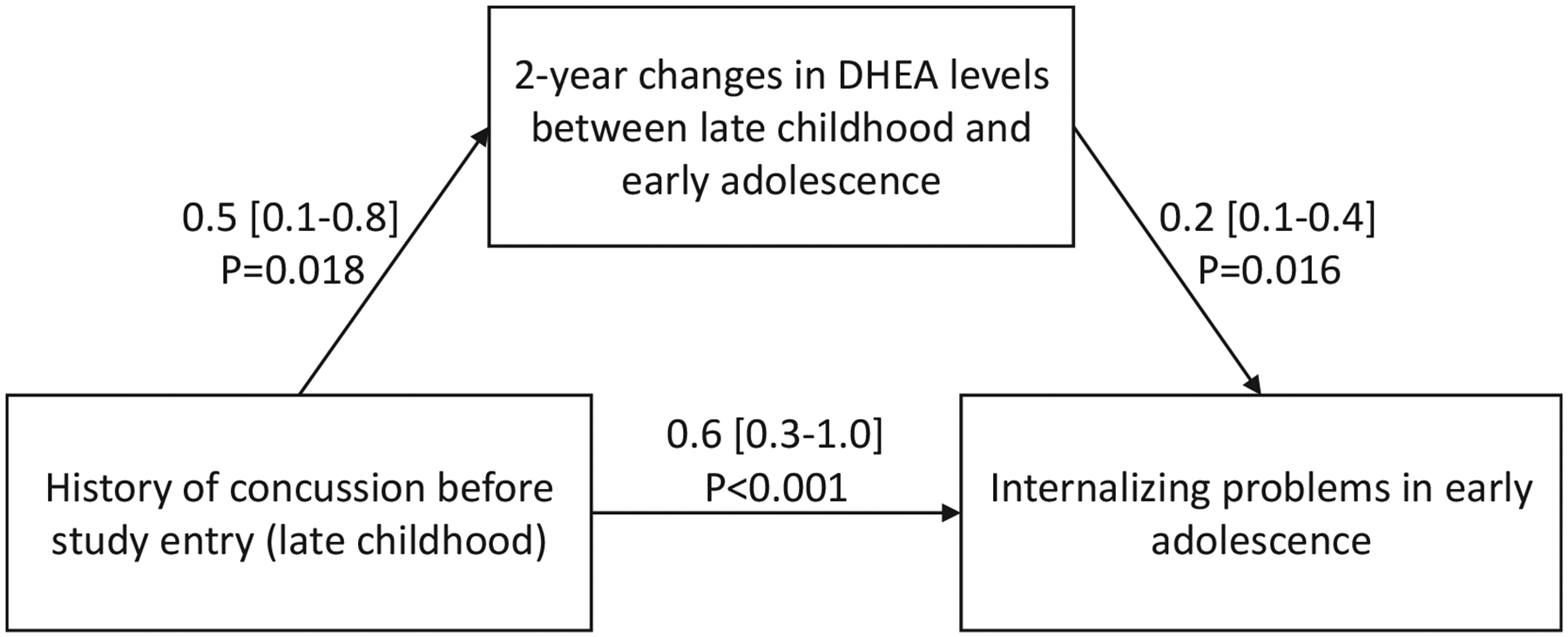
Mediation effect of 2-year changes of DHEA on the relationships between history of concussion and psychological health issues in early adolescence. The figure depicts the mediation model including history of concussion (independent Variable), 2-year changes in DHEA levels (mediator), and psychological health issues in early adolescence (dependent variable). Sample size: 54 participants with history of concussion and 58 participants with no history of concussion. The arrows connecting each component of the mediation model show the directionality of each relation, the regression coefficient, and the *p* value for the association. Psychological health issues in late childhood were a covariate in the model. DHEA = dehydroepiandrosterone; FDR = false discovery rate.

After accounting for levels of psychological issues in late childhood, history of concussion was associated with more severe psychological issues (F_[1,105]_ = 19.75, *p*< .001,Q < 0.001) in early adolescence. Mediation analyses revealed that this effect was partially mediated by the 2-year changes in DHEA (Figure 3). PROCESS macro demonstrated that larger increases in DHEA between late childhood and early adolescence partially mediated the effect of concussion on psychological issues (Figure 3). Specifically, history of concussion was associated with more severe psychological health issues in those who exhibited post-concussion increases in DHEA levels.

### Exploratory analyses

#### Contribution of demographic variables.

Rerunning 2.5.1 analyses after accounting for demographic and hormone collection confounders did not affect the main findings (Table S2).

#### Moderation analyses.

There was no significant moderation for the effect of concussion on pubertal hormones in late childhood (Table S3). Pubertal timing significantly moderated the effect of history of concussion on 2-year changes in testosterone (Table S3), with more advanced pubertal timing in late childhood being associated with a stronger effect of history of concussion on 2-year changes in testosterone. Yet, this effect did not survive multiple comparison correction. Furthermore, sex and pubertal timing moderated the association between history of concussion and psychological issues in late childhood and early adolescence, with girls and participants at a more advanced stage of pubertal maturity showing a stronger association between history of concussion and psychological issues (Table S4).

#### Externalizing problems.

History of concussion was associated with more severe externalizing problems in both late childhood (F_[1,257]_ = 22.85, *p* < .001, Q < 0.001) and early adolescence (F_[1,105]_ = 10.28, *p* = .001, Q = 0.001); however, no hormone mediated this relationship.

#### Indirect effects on pubertal maturation.

Low testosterone fully mediated the association between history of concussion and low PDSG scores at late childhood (direct effect = −0.15, SE = 0.12, confidence interval [CI] = [−0.38; 0.09]; indirect effect = −0.10, SE = 0.04, CI = [−0.015;−0.02]). Larger 2-year increases in DHEA fully mediated the association between history of concussion and higher PDSA scores at early adolescence (direct effect = −0.04, SE = 0.16, CI = [−0.34; 0.28]; indirect effect = 0.12, SE = 0.07, CI = [0.02; 0.26]).

#### Recency.

The time between concussion in the previous 2-year and study-entry did not correlate with levels of testosterone in late childhood (r_(130)_ = 0.1, *p* = .289) or changes in DHEA between late childhood and early adolescence (r_(52)_ = 0.2, *p* = .220).

## Discussion

This study investigated the effects of having a history of concussion on pubertal hormones measured in late childhood and early adolescence, and further determined the extent to which pubertal hormones can help explain the heightened risk for psychological health issues observed in pediatric concussion [1]. Partially consistent with our hypotheses, history of concussion was associated with lower levels of testosterone in late childhood; however, contrary to our hypothesis, history of concussion was associated with larger increase in DHEA from late childhood to early adolescence. Intriguingly, the DHEA increase, but not the lower testosterone, partially mediated the association between concussion and psychological issues in early adolescence.

Our finding of lower testosterone in children may reflect a concussion-related deficit in the hypothalamic–pituitary–gonadotropic (HPG) axis, which regulates the production of sex hormones across the lifespan [37]. In late childhood, the hypothalamus starts secreting gonadotropin-releasing hormone. Here, we speculate that a brain injury in late childhood could directly (e.g., impact and/or vascular damage) or indirectly (e.g., inflammation and/or stress response) [38] affect the functioning of the HPG axis. While these relationships remain understudied in younger groups, studies have shown structural and functional dysfunction involving the hypothalamus and pituitary gland following mild to severe brain injuries in adults [6–8]. Notably, our exploratory analyses showed that lower levels of testosterone in late childhood fully mediated the association between concussion and gonadal maturation, suggesting that experiencing a concussion as children transitioning into early adolescence might delay gonadarche and/or affect the development of secondary sexual characteristics through testosterone. While these findings should be interpreted with caution, the observed reduction (approximately 10% as shown in Figure 1) in testosterone is consistent with hormonally meaningful shifts seen during early pubertal development [30], highlighting the potential significance of these effects and the need for further prospective investigation. Considering the critical role of the HPG axis in regulating pubertal hormone production from late childhood [10—12], our findings suggest that a concussion during this period might impact early stages of puberty. However, a 2-year interval in puberty is a significant lapse. Prospective longitudinal studies with 3-to-6-month intervals are needed to clarify this relationship.

Contrary to our hypothesis, there were larger increases in DHEA between late childhood and early adolescence in those with (vs. those without) a history of concussion, and these changes were linked to faster adrenarcheal development in early adolescence. It is important to note that previous studies have shown that DHEA can be influenced by stress and illness [39]. The larger increases in DHEA and the advanced adrenarcheal maturation could also reflect a compensatory mechanism happening between concussion and hormone collection over time. This delayed association is consistent with the observation that physical changes lag behind hormonal changes during development [40]. Additionally, the larger increases in DHEA mediated the association between history of pediatric concussion and psychological health issues reported in adolescents with a history of concussion. Notably, we found that children with pediatric concussion also have more externalizing problems than those without such a history; however, it is important to note that pubertal hormones did not mediate this association. Consistent with our findings, research has suggested that faster pacing of development (e.g., faster tempo) is associated with a heightened risk for psychological issues [31]. Intriguingly, testosterone did not play a role in explaining the vulnerability for psychological issues in adolescents with a history of concussion, which may be associated with the fact that adrenarche usually starts before gonadarche and thus is more vulnerable to concussion during this period. While females tend to enter puberty earlier than males and report more psychological issues than males following a concussion, we did not observe a main effect of sex at birth on the relationship between concussion and pubertal hormones. In spite of the fact that we did not find that a history of concussion has a direct effect on gonadal hormonal changes (i.e., delta testosterone/estrogens), pubertal timing at baseline moderated the association between pediatric concussion and testosterone changes over time, suggesting that a concussion sustained in children who are more advanced in pubertal maturation may also affect gonadal hormones (and potentially gonadal maturation). Therefore, longitudinal studies in older samples will help understand the effect of concussion on later stages of puberty.

Our study involved a comprehensive and sizable dataset from the ABCD cohort, which allowed for broader generalizability of the findings and offered a unique take on the still understudied biological mechanisms of pediatric concussion and risk for psychological issues. However, concussion history was based on subjective parent recall, which may be biased. Additionally, the OSU-TBI was validated in adults with substance use disorders [24], which may limit its relevance for adolescents. In this study, we leveraged repeated hormonal measures in children with a history of concussion transitioning into early adolescence. As more data become available, this investigation should expand to mid and late adolescence. The lack of concussion-related findings on E2 may reflect limited power, particularly due to a smaller sample of females. Given the instrumental role of pubertal hormones on brain development, longitudinal studies using a combination of biomarkers (e.g., hormones, magnetic resonance imaging) are needed to examine how concussion affects the HPG axis, brain maturation, and psychological outcomes. Finally, while our findings focus on concussion, other important factors that influence psychological outcomes, such as family history of psychiatric illness, trauma, and/or sleep, were not included in our models due to relative small sample size and power limitations. Future research should use prospective, longitudinal designs that assess children shortly after concussion to distinguish acute from long-term effects on pubertal hormones. Larger samples with comprehensive assessments of concussion, hormonal influences (e.g., contraceptives, cortisol, supplements), and psychological health are needed to clarify these complex relationships and identify youth most at risk for poor outcomes.

### Conclusions

The current findings suggest that children with a history of concussion in childhood reported worse psychological health and had lower levels of testosterone and larger increases in DHEA during the transition from late childhood to early adolescence. These results provide preliminary evidence that pubertal hormones may play a role in the association between injury and risk for psychological health in adolescents. Importantly, these findings should not be seen as a reason to discourage participation in contact sports, as they provide numerous physical, social, and emotional benefits for young people. Instead, they emphasize the importance of careful monitoring and support after a pediatric concussion, especially during critical periods of neurobiological and hormonal development. Future longitudinal research is necessary to replicate these findings in prospective sample with concussion, investigate potential biological mechanisms, and elucidate the short- and long-term effects of pediatric concussion on pubertal hormones and normative development.

## Supplementary Material

1

Supplementary Data

Supplementary data related to this article can be found at https://doi.org/10.1016/j.jadohealth.2025.07.020.

## Figures and Tables

**Table 1:** Characteristics of the samples

Participants with data at late childhood				
Characteristics	Without history of concussion^a^ (N = 132)	With history of concussion (N = 132)	t_(262)_ or x^2^	*p* ^b^
Age (years), mean [SD]	9.87 [0.66]	10.03 [0.67]	t = −1.93	*.054*
Sex			x^2^ < 0.01	>.999
Male (%)	85 (64.39%)	85 (64.39%)		
Female (%)	47 (35.61%)	47 (35.61%)		
PDSS^c^, mean [SD]	1.71 [0.69]	1.77 [0.76]	t = − 0.60	.524
Pubertal timing^d^, mean [SD]	− 0.05 [0.99]	− 0.13 [0.92]	t = 0.71	.477
PDSA, mean [SD]	1.58 [0.93]	1.66 [0.96]	t = − 0.72	.474
PDSG, mean [SD]	2.01 [0.83]	1.88 [0.78]	t = 1.83	*.070*
Body mass index, mean [SD]	18.40 [3.41]	18.62 [4.07]	t = − 0.47	.637
Household income			x^2^ = 0.70	.413
Less than 50,000 (%)	41 (31.06)	34 (25.76%)		
Higher than 50,000 (%)	91 (68.94%)	98 (74.24%)		
Parent education			x^2^ = 5.28	**.022**
Lower education (%)	58 (43.94%)	39 (29.55%)		
Higher education (%)	74 (56.06%)	93 (70.45%)		
Race			x^2^ = 0.18	.673
White (%)	96 (72.73%)	100 (75.76%)		
Non-white (%)	36 (27.27%)	32 (24.24%)		
Time of collection since midnight (minutes), mean [SD]	765.97 [178.28]	755.38 [191.56]	t = 0.47	.642
Duration of collection (minutes), mean [SD]	7.13 [4.69]	6.61 [7.53]	t = 0.68	.499
Time between collection and freezer storage (minutes), mean [SD]	12.14 [24.62]	8.04 [10.93]	t = 1.75	*.083*
Intake of caffeine			x^2^ = 0.32	.569
Yes (%)	8 (6.06%)	5 (3.79%)		
No (%)	124 (93.94%)	127 (96.21%)		
Vigorous physical exercise			x^2^ = 0.34	.560
Yes (%)	17 (12.88%)	13 (9.85%)		
No (%)	115 (87.12%)	119 (90.15%)		
Participants with data at early adolescence				
Characteristics	Without history of concussion-concussed^a^ (N = 58)	With history of concussion (N = 54)	t (110) or x^2^	*p* ^b^
Age (years), mean [SD]	12.04 [0.66]	12.09 [0.67]	t = − 0.40	.679
Sex			x^2^ < 0.1	>.999
Male (%)	39 (67.24%)	36 (66.67%)		
Female (%)	19 (32.76%)	18 (33.33%)		
PDSS^e^, mean [SD]	2.53 [1.02]	2.38 [1.15]	t = 0.70	.487
Pubertal timing^d^, mean [SD]	0.06 [0.95]	− 0.07 [−0.90]	t = 0.70	.487
PDSA, mean [SD]	2.33 [1.06]	2.25 [1.16]	t = 0.38	.708
PDSG, mean [SD]	2.72 [1.22]	2.51 [1.28]	t = 0.88	.380
Body mass index, mean [SD]	19.81 [4.89]	20.05 [3.41]	t = − 0.31	.761
Household income			x^2^ = 1.00	.317
Less than 50,000 (%)	20 (34.48%)	13 (24.07%)		
Higher than 50,000 (%)	38 (65.52%)	41 (75.93%)		
Parent education			x^2^ < 0.1	>.999
Lower education (%)	17 (29.31%)	15 (27.78%)		
Higher education (%)	41 (70.69%)	39 (72.22%)		
Race			x^2^ = 0.55	.457
White (%)	44 (75.86%)	45 (83.33%)		
Non-white (%)	14 (24.14%)	9 (16.67.%)		
Time of collection since midnight (minutes), mean [SD]	804.33 [210.63]	771.87 [218.29]	t = 0.80	.425
Duration of collection (minutes), mean [SD]	6.88 [5.70]	7.13 [10.09]	t = − 0.16	.873
Time between collection and freezer storage (minutes), mean [SD]	9.00 [8.79]	9.31 [15.88]	t = − 0.13	.898
Intake of caffeine			x^2^ = 0.47	.494
Yes (%)	5 (9.43%)	2 (3.85%)		
No (%)	53 (90.57%)	52 (96.15%)		
Vigorous physical exercise			x^2^ = 0.17	.684
Yes (%)	9 (15.52%)	6 (19.23%)		
No (%)	49 (84.48%)	48 (80.77%)		

PDSA = pubertal development scale adrenal; PDSG = pubertal development scale gonadal; PDSS = pubertal development scale score; SD = standard deviation.

a Participants without history of concussion were selected from those typical developing adolescents (no past or current psychiatric disorders).

b *p* values ≤ 0.05 are reported in bold characters; *p* values between 0.05 and 0.10 are reported in *italics*.

c At study entry, 30 participants without a history of concussion and 40 with history of concussion did not start puberty (PDSS = 1). In addition, two participants without a history of concussion had PDSS = 5.

d Age-related effects were regressed-out from PDSS in male and female separately to derive pubertal timing for the analyses.

e In this sample, at study entry, 15 participants without a history of concussion and 19 with history of concussion did not start puberty (PDSS = 1). In addition, six participants without a history of concussion and 10 with history of concussion did not start puberty (PDSS = 1) by the follow-up. Finally, one participant without history of concussion had PDSS = 5 at study-entry and one participant in each group had PDSS = 5 at follow-up.

**Table 2:** Effects of history of concussion on levels of pubertal hormones and 2-year changes

Effects of history of concussion on levels of pubertal hormones in late childhood^a,b^			
Hormones	F	*p* ^c,d^	Q^c,d^
DHEA	F_(1,241)_ = 1.61	.201	0.302
Testosterone	F_(1,241)_ = 6.49	**.011**	**0.033**
Estradiol^e^	F_(1,73)_ = 0.03	.849	0.849
Effects of history of concussion on 2-year changes in levels of pubertal hormones^f,g^			
Hormones	F	*p* ^c,d^	Q^c,d^
DHEA	F_(1,90)_ = 6.11	**.015**	**0.045**
Testosterone	F_(1,90)_ = 0.	.724	0.724
Estradiol^e^	F_(1,19)_ = 3.83	*.065*	*0.098*

DHEA = dehydroepiandrosterone.

a Sample size: 132 participants with history of Concussion and 132 participants with no history of concussion. Analyses using raw Pubertal Development Scale Score instead of pubertal timing did not affect the findings.

b Covariates included parent education and Adolescent Brain Cognitive Development site.

c *p* values ≤ 0.05 are reported in bold characters and *p* values between 0.05 and 0.10 are reported in *italics*.

d Q represents the *p* values after false discovery rate correction.

e Only females were included in this analysis.

f Sample size: 54 participants with history of concussion and 58 participants with no history of concussion.

g The level in late childhood of the respective hormone was included as a covariate.
