# Supplementary material for: Connecting the Dots: The Role of Pediatric Concussion on Pubertal Hormones and Psychological Health
Source: J Adolesc Health. Author manuscript; Available in PMC 2026 Jul 15. (PMC13370396; doi:10.1016/j.jadohealth.2025.07.020)
Supplement: 1 [file NIHMS2191810-supplement-1.docx]

**SUPPLEMENTAL ONLINE CONTENT**

**Title:**

Connecting the Dots: The Role of Pediatric Concussion on Pubertal Hormones and Psychological Health

**List of components:**

Supplemental Material and Methods

Table S1. Hormones levels by sex.

Table S2. Re-assessing the effects of history of concussion on levels of pubertal hormones or 2-year changes after accounting for demographic and hormone collection confounders.

Table S3. Effect of age, pubertal timing, and sex on the association between history of concussion and pubertal hormones.

Table S4. Effect of age, pubertal timing, and sex on the association between history of concussion and psychological problems.

Figure S1. Inclusion and exclusion criteria flowchart.

Figure S2. Site distribution.

**Supplemental Material and Methods**

***Additional variables***

In the ABCD study, parent education, household income, and race/ethnicity were captured using multiple choices: 1. Parent education: Never attended/Kindergarten only; 1st grade; 2nd grade; 3rd grade; 4th grade; 5th grade; 6th grade; 7th grade; 8th grade; 9th grade; 10th grade; 11th grade; 12th grade; High school graduate; GED or equivalent; Some college; Associate degree: Occupational; Associate degree: Academic Program; Bachelor's degree ; Professional School degree; Master Degree; Bachelor’s Degree; Doctoral Degree.; 2. Household income: Less than $5,000; $5,000 through $11,999; $12,000 through $15,999; $16,000 through $24,999; $25,000 through $34,999; $35,000 through $49,999; $50,000 through $74,999; $75,000 through $99,999; $100,000 through $199,999; $200,000 and greater; and 3. Race/ethnicity: White, Black, Hispanic, Asian, and other. However, due to the smaller sample in this work relative to the original ABCD study, parent education was categorized into two groups (lower or higher education than bachelor’s degree), household income into two groups (income less than $50k; income greater than $50k), and race/ethnicity into two groups (white and non-white).

**Supplemental tables**

**Table S1. Hormones levels by sex.**

| Late childhood data (N=264 participants) | | | | | |
| --- | --- | --- | --- | --- | --- |
| Hormone (pg/ml) | History of Concussion | Male | Female | Statistic | P^a^ |
| Testosterone, mean [SD] | Yes | 26.59 [11.60] | 31.71 [13.95] | t(130) = 2.1 | **0.035** |
|  | No | 29.92 [12.32] | 36.83 [15.01] | t(130) =2.7 | **0.009** |
| DHEA, mean [SD] | Yes | 45.49 [29.86] | 62.61 [34.87] | t(130) = 2.8 | **0.006** |
|  | No | 51.72 [31.28] | 64.49 [35.48] | t(130) = 2.1 | **0.042** |
| Early adolescence data (N=112 participants) | | | | | |
| Hormone (pg/ml) | History of Concussion | Male | Female | Statistic | P^a^ |
| Testosterone, mean [SD] | Yes | 53.73 [25.03] | 52.98 [20.72] | t(56) = -0.1 | 0.908 |
|  | No | 55.44 [35.86] | 49.34 [20.76] | t(42) = -0.8 | **0.042** |
| DHEA, mean [SD] | Yes | 69.84 [42.08] | 113.18 [83.45] | t(56) = 2.1 | 0.051 |
|  | No | 62.44 [56.38] | 88.83 [58.15] | t(52) = 1.7 | 0.110 |

^a^ P values ≤ 0.05 are reported in **bold** characters.

**Table S2. Re-assessing the effects of history of concussion on levels of pubertal hormones or 2-year changes after accounting for demographic and hormone collection confounders.**

| Effects of history of concussion on levels of pubertal hormones in late childhood.^a^ | | | |
| --- | --- | --- | --- |
| Hormones | F | P^b,c^ | Q^b,c^ |
| DHEA | F_(1,231) =_ 1.57 | 0.216 | 0.317 |
| Testosterone | F_(1,231) =_ 6.73 | **0.010** | **0.030** |
| Estradiol^d^ | F_(1,64) =_ 0.04 | 0.847 | 0.847 |
| Effects of history of concussion on 2-year changes in the levels of pubertal hormones.^e^ | | | |
| Hormones | F | P^b,c^ | Q^b,c^ |
| DHEA | F_(1,80) =_ 6.12 | **0.016** | **0.046** |
| Testosterone | F_(1,80) =_ 0.90 | 0.345 | 0.345 |
| Estradiol^d^ | F_(1,10) =_ 3.74 | *0.082* | 0.123 |

Abbreviations: DHEA – Dehydroepiandrosterone.

^a^ Sample size: 132 participants with history of Concussion and 132 participants with no history of concussion. Covariates included: Sex, pubertal timing, body mass index (BMI), race, household income, parent education, duration of collection (minutes), intake of caffeine, time between collection and freezer storage (minutes), time of collection since midnight (minutes), vigorous physical exercise, and ABCD site. Analyses using raw PDSS did not affect the findings.

^b^ P values ≤ 0.05 are reported in **bold** characters and P values between 0.05 and 0.10 are reported in *italics*.

^c^ Q represents the P values after False Discovery Rate (FDR) correction.

^d^ Only girls were included in this analysis.

^e^ Sample size: 54 participants with history of Concussion and 58 participants with no history of concussion. Covariates included: Sex, pubertal timing, body mass index (BMI), race, household income, parent education, duration of collection (minutes), intake of caffeine, time between collection and freezer storage (minutes), time of collection since midnight (minutes), vigorous physical exercise, and ABCD site. The level at late childhood of the respective hormone was included as an additional covariate. Analyses using raw PDSS did not affect the findings.

**Table S3. Effect of age, pubertal timing, and sex on the association between history of concussion and pubertal hormones.**

| Late childhood hormones^a^ | Moderators | | | | | | | | |
| --- | --- | --- | --- | --- | --- | --- | --- | --- | --- |
|  | Age | | | Pubertal timing | | | Sex | | |
|  | F | P^b^ | Q^b,c^ | F | P^b^ | Q^b,c^ | F | P^b^ | Q^b,c^ |
| DHEA | F_(1,239)_ = 0.77 | 0.382 | 0.382 | F_(1,239)_ = 0.30 | 0.587 | 0.587 | F_(1,239)_ = 0.36 | 0.549 | 0.591 |
| Testosterone | F_(1,239)_ =1.81 | 0.179 | 0.2685 | F_(1,239)_ = 0.58 | 0.448 | 0.587 | F_(1,239)_ = 0.29 | 0.591 | 0.591 |
| Estradiol^d^ | F_(1,73)_ = 3.38 | *0.070* | 0.210 | F_(1,73)_ = 0.31 | 0.576 | 0.587 | - | - | - |
| 2-year changes^e^ | Moderators | | | | | | | | |
|  | Age | | | Pubertal timing | | | Sex | | |
|  | F | P^b^ | Q^b,c^ | F | P^b^ | Q^b,c^ | F | P^b^ | Q^b,c^ |
| DHEA | F_(1,89) =_ 0.04 | 0.834 | 0.834 | F_(1,89) =_ 1.16 | 0.284 | 0.284 | F_(1,89) =_ 1.26 | 0.264 | 0.264 |
| Testosterone | F_(1,89) =_ 0.52 | 0.474 | 0.834 | F_(1,89) =_ 5.53 | **0.021** | *0.063* | F_(1,89) =_ 1.55 | 0.217 | 0.264 |
| Estradiol^d^ | F_(1,18) =_ 0.08 | 0.776 | 0.834 | F_(1,18) =_ 2.56 | 0.120 | 0.18 | - | - | - |

Abbreviations: DHEA – Dehydroepiandrosterone.

^a^ Sample size: 132 participants with history of Concussion (47 female) and 132 participants with no history of concussion (47 female).

^b^ P values ≤ 0.05 are reported in **bold** characters.

^c^ Q represents the P values after False Discovery Rate (FDR) correction. P values between 0.05 and 0.10 are reported in *italics*.

^d^ Moderation analyses with sex as a moderator could not be performed given that estradiol was only available in girls.

^e^ Sample size: 54 participants with history of Concussion (18 female) and 58 participants with no history of concussion (19 female).

**Table S4. Effect of age, pubertal timing, and sex on the association between history of concussion and psychological problems.**

| Moderator | Late childhood^a^ | | | Early adolescence^b^ | | |
| --- | --- | --- | --- | --- | --- | --- |
|  | F | P^c^ | Q^c,d^ | F | P^c^ | Q^c,d^ |
| Age | F_(1,239)_ < 0.01 | 0.948 | 0.948 | F_(1,89)_ = 0.01 | 0.905 | 0.905 |
| Pubertal timing | F_(1,239)_ = 8.02 | **0.005** | **0.015** | F_(1,89)_ = 10.89 | **0.001** | **0.003** |
| Sex | F_(1,239_) = 5.01 | **0.026** | **0.039** | F_(1,89)_ = 7.65 | **0.007** | **0.011** |

^a^ Sample size: 132 participants with history of Concussion (47 female) and 132 participants with no history of concussion (47 female).

^b^ Sample size: 54 participants with history of Concussion (18 female) and 58 participants with no history of concussion (19 female).

^c^ P values ≤ 0.05 are reported in **bold** characters.

^d^ Q represents the P values after False Discovery Rate (FDR) correction. P values between 0.05 and 0.10 are reported in *italics*.

**Supplemental Figures**

**Figure S1: Inclusion and exclusion criteria flowchart.**


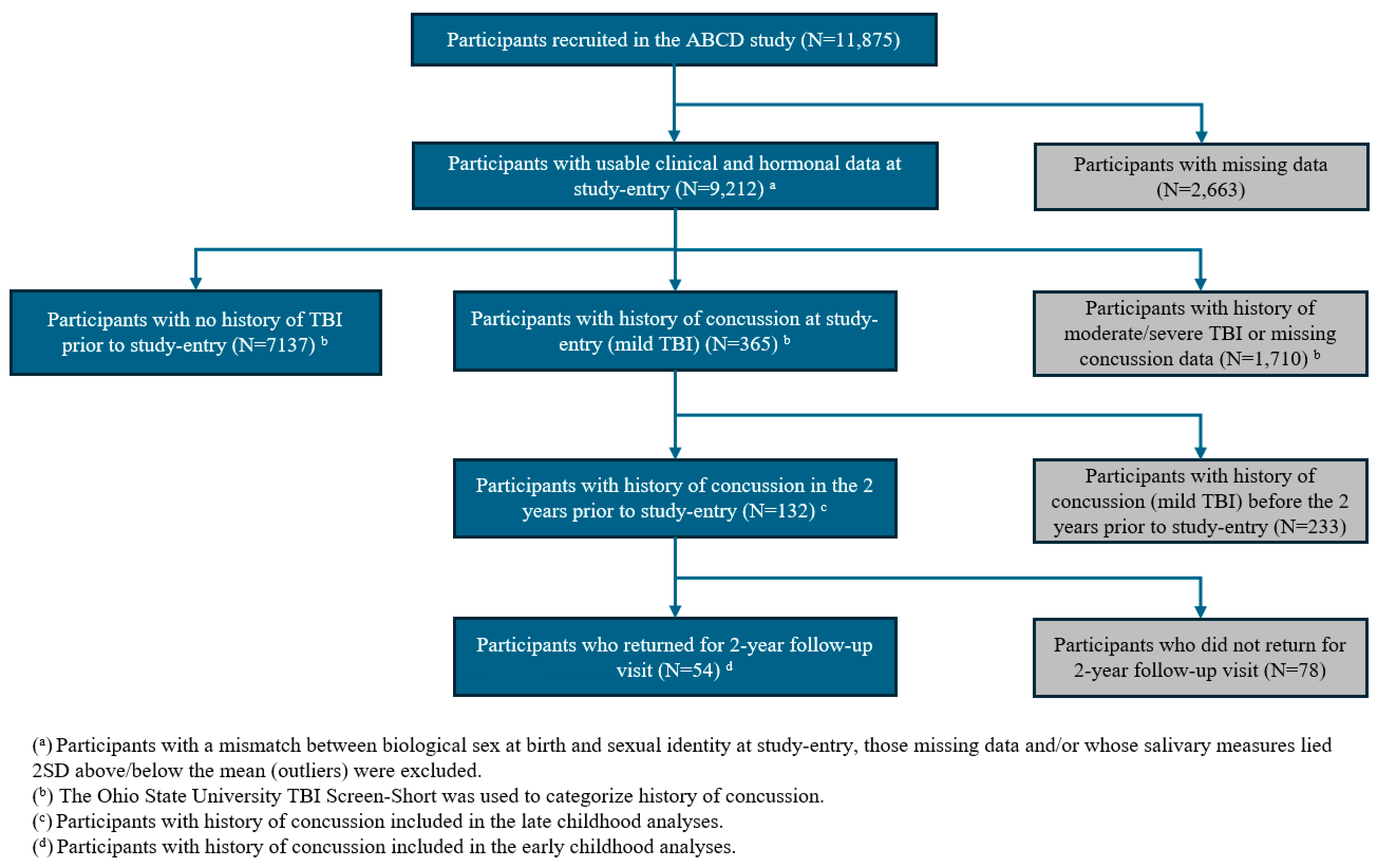


**Figure S1 legend:** Figure S1 shows the inclusion and exclusion criteria applied in this study. Blue boxes indicate the participants meeting the inclusion criteria and gray boxes indicate participants excluded from the final sample.

**Figure S2:** **Site distribution.**


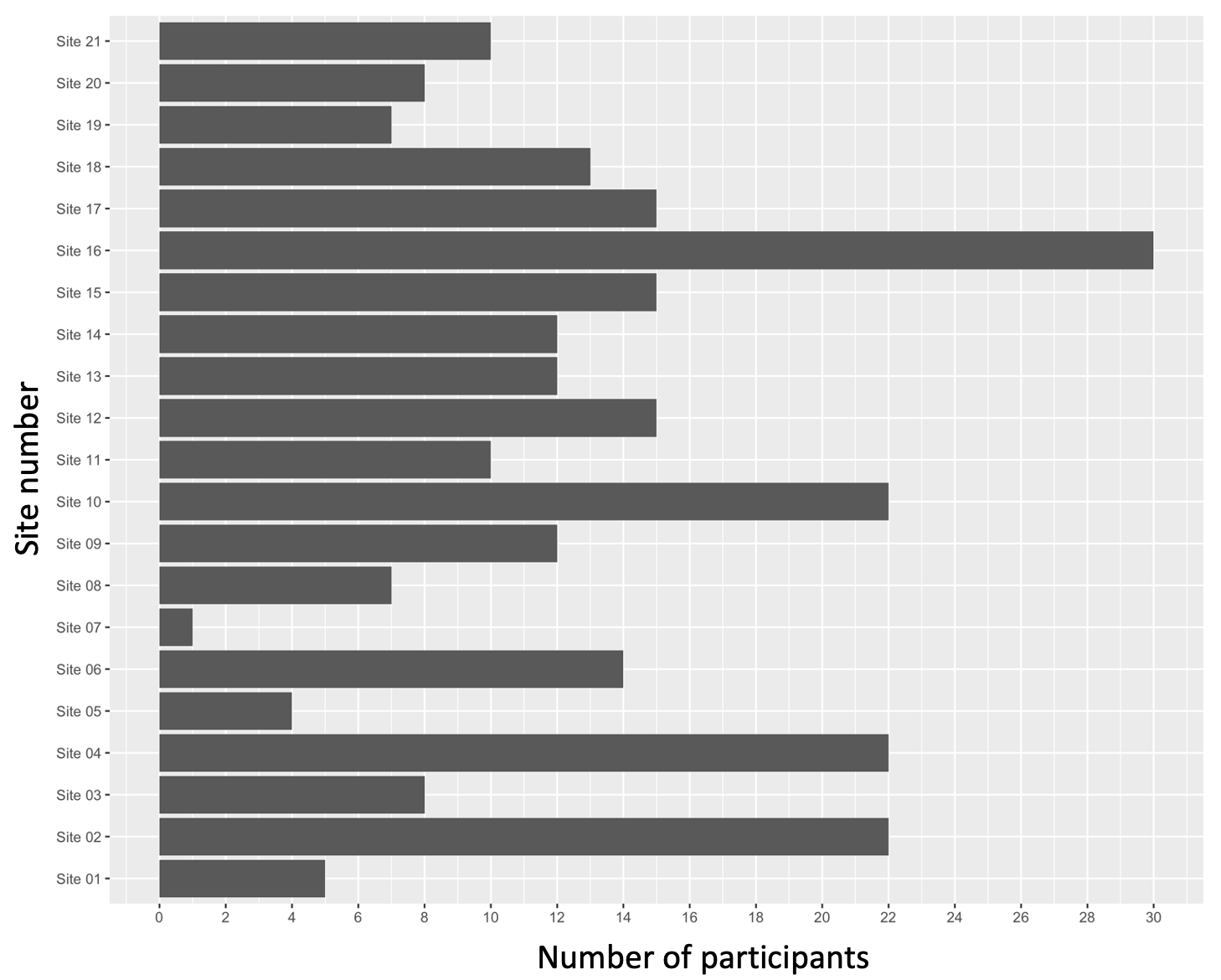


**Figure S2 legend:** Figure S2 shows the site distribution of the participants included in the analyses. The x-axis shows the number of participants, and the y-axis shows the number of participants in each site.
